# Is PESI a reliable tool for predicting early mortality in acute pulmonary embolism? Real-life evidence from a single-center study

**DOI:** 10.36416/1806-3756/e20250122

**Published:** 2025-11-14

**Authors:** Tugce Karamustafalioglu, Sibel Nayci, Yuksel Balci, Eylem Sercan Ozgur

**Affiliations:** 1. Department of Pulmonology, Medical Faculty Hospital, Mersin University, Mersin, Turkey.; 2. Department of Radiology, Medical Faculty Hospital, Mersin University, Mersin, Turkey.

## TO THE EDITOR,

Recent publications on pulmonary thromboembolism (PTE) have reported signs of right ventricular dysfunction (RVD) on echocardiography or computed tomography (CT) in approximately 34% of patients classified as low risk according to the Pulmonary Embolism Severity Index (PESI).^1^ However, these patients are not represented in the early mortality risk assessment tables of current guidelines.^2^ ^,^ ^3^ We defined this subgroup as the so-called “missing group” and aimed to evaluate their clinical characteristics and 30-day mortality outcomes, given the uncertainty surrounding their risk classification.

Between 2018 and 2023, patients diagnosed with PTE at our Pulmonology Clinic were screened to identify individuals meeting the criteria for the “missing group”. We analyzed data from patients with low-risk PESI and RVD, representing a rare and underexplored subgroup.

In the present study, patients with <86 points on the PESI (i.e., Class I or II, defined as low risk) were assessed for radiologic and echocardiographic signs of RVD.^4^ On chest CT, the following findings were considered markers of RVD: pulmonary artery/aorta diameter ratio ≥1, pulmonary artery diameter ≥3 cm, presence of contrast material in the inferior vena cava (IVC), and right ventricular/left ventricular diameter ratio ≥1.^3^ Echocardiographic markers included RV dilation (RV/LV ratio >1), RV hypokinesia, interventricular septal flattening, elevated systolic pulmonary artery pressure, and a dilated IVC with diminished respiratory collapse.^3^

The anatomical extent and location of thrombi were quantified using the Pulmonary Arterial Obstruction Index (PAOI), as modified by Qanadli et al.,^5^ which considers segmental involvement of the pulmonary arterial tree and assigns points accordingly. 

Twenty-two of the 672 PTE patients (3.2%) met the inclusion criteria for the missing group (Table 1). Their mean age was 44 ± 10 years, and 4 were female (18.2%). A total of 18 patients (81.8%) had no comorbidities. The 30-day mortality rate in this subgroup was 9% (2 patients). One of the deceased was a 29-year-old female (PESI 0, sPESI 0; the simplified Pulmonary Embolism Severity Index [sPESI] is a six-variable scoring system-age >80 years, history of cancer, chronic cardiopulmonary disease, heart rate ≥110 bpm, systolic blood pressure ≤100 mmHg, and oxygen saturation <90%-in which scores ≥1 indicate a higher risk of early mortality), and the other was a 47-year-old male (PESI 10, sPESI 1); both presented with chest pain. One patient had a history of massive pulmonary embolism 11 years earlier, whereas the other had no comorbidities. Their vital signs showed only mild tachycardia (105 and 102 bpm) and slightly reduced oxygen saturation (92% and 91%, respectively). Both had RVD on chest CT and elevated troponin levels. One patient died in hospital, and the other died 11 days post-discharge. These findings highlight a potential gap in risk stratification for patients who appear low risk based on PESI/sPESI but still experience adverse outcomes.

**Table 1:** Classification of 22 patients based on right ventricular overload findings.

		ECHO or CT		Troponin	Qanadli Index Average
	n	ECHO	CT		
If the PESI were high, these patients would be in the “intermediate-high” risk group (n = 13)	9	+	+	+	63.8%
	1	+	-	+	27.5%
	3*	-	+	+	25%
If the PESI were high, these patients would be in the “intermediate-low” risk group (n = 9)	1	-	-	+	17.5%
	6*	+	+	-	41.6%
	0	-	+	-	
	2	+	-	-	26.2%

*The deceased patients belonged to these groups. CT findings = RV/LV ≥1 and/or PA diameter ≥3 cm and/or PA/AO diameter ratio ≥1 and/or presence of contrast agent in the IVC. Abbreviations: PESI, Pulmonary Embolism Severity Index; CT, computed tomography; ECHO, echocardiography; RV, right ventricle; LV, left ventricle; PA, pulmonary artery; AO, aorta; IVC, inferior vena cava.

The PESI is a scoring system developed using parameters such as demographic characteristics, comorbidities, and vital signs. It is applied to distinguish intermediate- from low-risk groups in the early mortality risk assessment table. Despite its important role, the PESI has some limitations. For example, patients receive points only when oxygen saturation falls below 90%. However, in younger patients, values of 91-92% should also be considered low and warrant closer monitoring.^6^ One of our deceased patients had an oxygen saturation of 91%. Because this value was above the cutoff, the patient’s PESI was classified as low, yet death occurred 3 days after the diagnosis. In addition, a history of previous PTE is not among the PESI parameters. The other deceased patient had a history of massive PTE 11 years earlier, but because this factor is not considered in the scoring system, the patient’s PESI was also low. Nevertheless, this patient died within 30 days.

For patients diagnosed with PTE and classified as intermediate risk in the early mortality risk assessment table, the 30-day mortality rate ranges from 5% to 15%.^3^ In our missing group, a 9% mortality rate was observed, which is notable despite the limited sample size. This finding suggests that careful reassessment of early mortality risk stratification tools is warranted.

This study has two limitations that should be addressed in future research. First, its retrospective design. Second, although the sample size was modest, it was sufficient to provide preliminary insights. The strength of this work lies in its focus on a rarely characterized subgroup within the PTE population. Larger, prospective studies are needed to confirm and expand upon these findings.

Although the Qanadli index was calculated for all patients who underwent chest CT, it did not correlate directly with early mortality in our cohort. The two patients who died had only moderate obstruction scores, suggesting that clot burden alone may not be sufficient for risk assessment. Our results emphasize the importance of integrating anatomical assessment with biomarkers and RVD evaluation to improve prognostication.

Only a small proportion of patients diagnosed with PTE present with both RVD and a low PESI. Although current guidelines mention this condition either in the text or in table footnotes, it is not included in the most widely used and practical early mortality risk assessment tables. This omission may contribute to under-recognition in clinical practice. Our findings raise the question of whether the PESI alone is sufficient to guide management in all cases.

In light of these concerns, our findings underscore the need for an updated and more comprehensive risk stratification tool. Such a system should integrate elements from other validated instruments, such as the Hestia score-which incorporates recurrence risk and social considerations-and include clinical history, particularly prior venous thromboembolism episodes. This approach may more effectively identify high-risk patients who might otherwise be overlooked by traditional metrics.

Thank you for the opportunity to share our perspective.
